# Supplementary material for: Content-rich biological network constructed by mining PubMed abstracts
Source: BMC Bioinformatics. 2004 Oct 8;5:147. doi: 10.1186/1471-2105-5-147 (PMC528731; doi:10.1186/1471-2105-5-147)
Supplement: Additional File 2 — The original results of the above study (non-essential files are deleted to keep the file size under the limit set by BMC bioinformatics). [file 1471-2105-5-147-S2.bz2 › chilibotAdditionalFile2/dip05/79ID9033409E291/html/CDC42_PAK3.html]

 


 **CDC42** and **PAK3** 
  
Found 226 abstracts in PubMed, retrieved 05.  
 

 What does Google say? 
 PDF only 
| .edu only 

---

**Interactive relationship** (e.g. stimulation, inhibition, etc)

**Neutral relationship**- p21  [ **PAK3** ]  activated protein kinases PAK are a family of serine threonine protein kinases which are activated by binding of the p21  [ **PAK3** ]  G proteins  **Cdc42**  or Rac.  Ref: 12853446 J Biol Chem< MedlineTA>J Biol Chem,
- The p21  [ **PAK3** ]  activated protein kinases Paks are serine threonine protein kinases activated by binding to Rho family small GTPases, Rac and  **Cdc42** .  Ref: 12860998 J Biol ChemJ Biol Chem,

**Non-interactive relationship** (e.g. studied together, co-existance, homology, etc.)

- Mutation of histidine residues 19 and 22 to leucine on the p21  [ **PAK3** ]  binding domain of Pak5 completely abolished the binding of  **Cdc42**  and the  **Cdc42**  mediated autophosphorylation.  Ref: 12860998 J Biol ChemJ Biol Chem,
- We describe a signaling mechanism involving Gbetagamma, PAK associated guanine nucleotide exchange factor PIXalpha,  **Cdc42** , and p21  [ **PAK3** ]  activated kinase PAK 1.  Ref: 12887923 Cell, 2003
- The p21  [ **PAK3** ]  activated kinase PAK homolog, Shk1, is a critical component of a multi functional Ras  **Cdc42**  PAK complex required for viability, polarized growth and cell shape, and sexual differentiation in the fission yeast, Schizosaccharomyces pombe.  Ref: 12764130 J Biol Chem< MedlineTA>J Biol Chem, 2003
